# Characteristics of chlorophyll fluorescence in the light‐adapted state as indicators of plant leaf relative water content

**DOI:** 10.1002/aps3.70090

**Published:** 2026-11-05

**Authors:** Junqing Chen, Lijiang Fu, Ya Guo, Felix B. Fritschi, Jinglu Tan

**Affiliations:** ^1^ Department of Chemical and Biomedical Engineering University of Missouri Columbia Missouri USA; ^2^ School of IOT Jiangnan University Wuxi Jiangsu China; ^3^ Division of Plant Science and Technology University of Missouri Columbia Missouri USA

**Keywords:** chlorophyll fluorescence (ChlF), nonphotochemical quenching (NPQ), PSII quantum efficiency, relative water content (RWC), steady‐state fluorescence (*F_s_*)

## Abstract

**Premise:**

Chlorophyll fluorescence (ChlF) in the light‐adapted state is a convenient indicator of plant water status but is heavily reliant on nonphotochemical quenching (NPQ). The usefulness of ChlF as an indicator can be enhanced by reducing NPQ effects.

**Methods:**

ChlF and relative water content (RWC) were measured from leaves of *Arabidopsis thaliana* and *Spinacia oleracea* while the leaf samples progressively lost water. ChlF characteristics were then reformulated and tested as indicators of RWC via regression analysis.

**Results:**

We demonstrate that measured NPQ varies and is by itself not a consistent indicator of RWC of plant leaves. Using NPQ as a scaler in ChlF‐based photochemical efficiency measures resulted in two NPQ‐adjusted ChlF characteristics as indicators of RWC: photosystem II photochemical quenching (PSII PQ) and an NPQ‐scaled PSII photochemical efficiency (Y(II)*′*) measure. The results were experimentally demonstrated in *A. thaliana*, then validated in *A. thaliana* with varied illumination intensities, and finally validated in another plant (*S. oleracea*). Further analysis revealed a linear correlation between steady‐state ChlF (*F*_*s*_) and maximum fluorescence (*F*_*m*_ *′*) across different RWCs and a time constant to account for the NPQ effect.

**Discussion:**

This research shows that ChlF characteristics are affected by NPQ during water loss and that adjusting PSII efficiency measures based on NPQ provides enhanced effectiveness in indicating plant water status.

Reliable and convenient methods to measure plant water status are of great interest because of the essential nature of water in plant growth and development, including its crucial role in photosynthetic metabolism (Dastur, 1925). Leaf tissue water status can significantly impact photosynthesis through reduced energy conversion caused by reduced gas exchange, chlorophyll content variation, and decreased enzyme activity (Sengupta et al., 2013). Chlorophyll fluorescence (ChlF) techniques are widely used to investigate photochemical reactions for insights into photochemical and nonphotochemical yields (Roháček, 2002; Murchie and Lawson, 2013; Guo and Tan, 2015); they are also commonly used as rapid and cost‐effective methods for assessing plant responses to water deficit stress (Bresson et al., 2015).

Various ChlF characteristics or ChlF‐based measures are commonly evaluated in the analysis of photosystem II (PSII) performance based on the three energy pathways (Maxwell and Johnson, 2000; Ibaraki and Murakami, 2006; Lazár, 2015): photochemical quenching, nonphotochemical quenching (NPQ), and fluorescence emission. The energy transfer rates for these pathways are represented by rate constants: *k*_*P*_ for photochemical quenching, *k*_*N*_ for NPQ, and *k*_*F*_ for fluorescence. Additionally, thermal dissipation independent of light‐induced processes is represented by the rate coefficient *k*_*D*_. These rates are used to define different measures of photoenergy transfer such as photochemical quantum efficiency or yield of PSII (Y(II) or Φ_PSII_), electron transport rate (ETR) in photochemical quenching, and NPQ (Porcar‐Castell, 2011), which are calculated from different ChlF measurements (Ritchie and Bunthawin, 2010). Specifically, to determine Y(II), ETR, and NPQ, saturating pulses of light are applied on dark‐adapted leaves to yield a maximum fluorescence (*F*_*m*_) after a minimum fluorescence (*F*_0_) is measured and also applied on light‐adapted leaves to obtain a maximum light‐adapted fluorescence (*F*_*m*_ *′*) during a period of steady‐state fluorescence (*F*_*s*_). The difference between the maximum and minimum is termed variable fluorescence (abbreviated as *F*_*v*_ for dark adapted or as *F*_*v*_ *′* for light adapted).

The maximum quantum efficiency of PSII in dark‐ and light‐adapted states (measured as *F*_*v*_ */F*_*m*_ and *F*_*v*_ *′/F*_*m*_ *′*, respectively), electron transport rate (ETR(II) = α × β × Y(II) × PAR, with α, β, and PAR being leaf absorbance, fraction of photons partitioned to PSII, and photosynthetically active radiation, respectively), and NPQ (measured as (*F*_*m*_ − *F*_*m*_ *′*)/*F*_*m*_ *′*) have been used as indicators of drought stress (Castañeda‐Murillo et al., 2022). Dark‐adapted *F*_*v*_ */F*_*m*_ can reflect the effects of photoinhibition and plant diseases (Xia et al., 2023); however, it tends to remain unchanged during short‐term drought and its correlation with drought stress is closely linked to chlorophyll content (Li et al., 2019). Moreover, it was recently found that *F*_*v*_ */F*_*m*_ cannot effectively represent the efficiency of PSII photochemistry (Sipka et al., 2021), pointing to the need for measures in the light‐adapted state. ETR(II) provides insights into the efficiency of PSII in converting absorbed photoenergy into chemical energy (Habermann et al., 2019). Because α and β in the ETR calculation often do not vary much and are often taken as constants in the literature (0.84 and 0.5, respectively), ETR(II) is usually proportional to Y(II) for a given PAR or actinic light. Y(II) (measured as (*F*_*m*_ *′* − *F*_*s*_)/*F*_*m*_ *′* = *F*_*v*_ *′*/*F*_*m*_ *′*), as the PSII quantum efficiency in the light‐adapted state and part of ETR(II), decreases with increasing water deficit stress (Pereira et al., 2019). A reduction in water molecules used in photochemical quenching is known to lower the overall efficiency of PSII (Kannan and Kulandaivelu, 2011; Ashraf and Harris, 2013); however, many factors and variables affect the photosynthetic system (Ruban, 2015, 2016) and thus the quantum efficiency may not most effectively reflect plant water content.

Because NPQ, photochemical quenching, and ChlF account for the total photoenergy absorbed, the extent of NPQ must influence ChlF characteristics and consequently the use of ChlF characteristics as indicators of photochemical reactions under light‐adapted conditions (Zhang et al., 2019). Notably, NPQ has been shown to have a positive correlation with moisture loss during short‐term drought (Li et al., 2008; Zivcak et al., 2013; Lima Neto et al., 2017; Ghorbanzadeh et al., 2021). Drought‐tolerant plants exhibit higher NPQ, indicating a protective mechanism whereby these plants dissipate more heat for self‐preservation (Colom and Vazzana, 2003; Bano et al., 2021). It is important to note, however, that the correlation between NPQ and tissue water content may not remain consistently strong, especially when drought conditions persist for an extended period. Furthermore, NPQ levels may decrease after a period of drought because of reductions in chlorophyll content (Csintalan et al., 1999; Da Silva and Arrabaca, 2004; Singh et al., 2014). In addition, leaf developmental stage is a factor in NPQ, with drought having a limited impact on the NPQ of younger leaves but leading to a significant reduction in NPQ for older leaves (Xu et al., 2008). Variations in NPQ, if unaccounted for, can directly increase variations in ChlF characteristics and therefore reduce the usefulness of ChlF‐based measures, such as Y(II) and ETR(II), as indicators of plant water status.

In this research, after demonstrating that strong sample‐to‐sample variations in NPQ make NPQ an inconsistent indicator of relative water content (RWC), we showed that accounting for the influence of NPQ in ChlF‐based PSII quantum efficiency measures enhances their effectiveness as indicators of plant leaf water status. Because RWC is closely related to photochemical activities, whereas NPQ can vary among samples because of anatomical and physiological differences, adjusting for NPQ can improve the robustness of ChlF‐based indicators of plant water status. This concept was demonstrated experimentally with different plant species and varied actinic light intensities.

## METHODS

### Plant samples

*Arabidopsis thaliana* (L.) Heynh. was grown in a growth chamber (temperature 18°C, lighting at 200 μmol/m^2^/s for 8 h per day, relative humidity at 30%), while *Spinacia oleracea* ‘Correnta’ was grown in a greenhouse in November and December (temperature 16–21°C, natural sunlight at 3.5–3.9 kWh/m^2^/d for 10–11 h a day, relative humidity at 15–20%). Seeds of *A. thaliana* ecotype IS3731 and *S. oleracea* were sown in 15.2‐cm and 30.4‐cm pots, respectively, filled with Miracle‐Gro media/soil and nitrogen fertilizer (Scotts Miracle‐Gro Company, Marysville, Ohio, USA), and were grown under well‐watered conditions for three and five weeks, respectively, prior to utilization. Five plants representing five replications were grown for each species. Each sample thus refers to a separate plant.

### Fluorescence measurement

ChlF was measured with a chlorophyll fluorometer (model OS5p+; Opti‐Sciences, Hudson, New Hampshire, USA). All samples underwent a 30‐min dark adaptation before every pulse amplitude modulation (PAM) measurement. The minimum ChlF (*F*_0_) was measured after dark adaptation followed by the measurement of the maximum ChlF (*F*_*m*_) with a 1‐s‐long saturating pulse of 5000 μmol/m^2^/s and actinic light of 1000 μmol/m^2^/s. After illumination by actinic light for 8 min, the steady‐state ChlF (*F*_*s*_) was measured, and saturating pulses were applied to obtain the maximum ChlF under light‐adapted conditions (*F*_*m*_ *′*). When the *F*_*m*_ *′* values generated by three pulses were within 3%, a steady light‐adapted state was considered reached. After removing the actinic illumination, the minimum ChlF after light adaptation was measured (*F*_0_′). The ChlF measurement interval was 10 ms during saturating pulses and 1 s during constant actinic illumination.

### Leaf excision, dehydration, and determination of leaf relative water content

To examine the relationship between ChlF and leaf RWC, one uppermost mature leaf of visually similar color and size was excised from each plant at 19:00 hours and immediately submerged in 22°C deionized water for 8 h at room temperature under darkness to attain turgid weight. Each leaf sample was used for ChlF measurements at progressively lower RWCs. RWC was calculated as: (sample weight − dry weight)/(turgid weight − dry weight) × 100% based on determination of mass by using a precision balance (model PX323/E; OHAUS, Parsippany, New Jersey, USA). The turgid weight was measured before dark adaptation, and the sample weight was measured immediately after each ChlF measurement. The dry weight was measured after drying in an oven at 40°C for 5 h. Between measurements, the leaf samples were placed in standard room conditions (22°C, 30% relative humidity, fluorescent lighting). ChlF and leaf mass were measured on each sample leaf at varied RWC values as it progressively lost water from turgid status. While the moisture loss process was not actively regulated, the RWC varied with time in a similar rate and fashion among the excised leaves.

### Data analysis

ChlF characteristics as measurable indicators of RWC were assessed and compared according to their dependence on or correlation with RWC. Regression analysis was performed and the *R* ^2^ value was used as the measure of dependence or correlation. Pairs of measured ChlF characteristics and RWC values from the five sample plants of each plant species were pooled for regression analysis in MATLAB (version R2018a; MathWorks, Natick, Massachusetts, USA). A *p*‐value of regression less than 0.05 was considered as significant dependence. Analysis of variance was performed in MATLAB at a significance level of α = 0.05 to measure differences among replicate sample plants.

## RESULTS

The measurement results are presented first to demonstrate the significant sample‐to‐sample variation in NPQ, which makes NPQ an inconsistent indicator of leaf RWC. ChlF‐based measures were then formulated to reduce the variable effects of NPQ based on light‐adapted measurements from *A. thaliana* samples. These proposed measures were further validated by using data acquired under varied light intensities or from another species (*S. oleracea*). Finally, an analysis of other NPQ‐related variables is provided.

### Sample‐to‐sample NPQ variations

NPQ values varied among samples grown under identical conditions or even from the same plant. ChlF responses of five initially dark‐adapted fresh *A. thaliana* samples, all at 99% RWC without other treatments, to an actinic light of 1000 μmol/m^2^/s and four saturating pulses (one early for *F*_*m*_ measurement and three in steady state for *F*_*m*_ *′* measurement) are shown in Figure 1. At the end of the last pulse, the actinic lighting was turned off, causing ChlF to decrease, in order to obtain *F*_0_′. Although the samples were grown under the same conditions and at the same RWC, their ChlF responses and NPQ values were different, apparently as a result of different ChlF behaviors among the samples. For example, sample 2 in Figure 1 exhibited a faster ChlF drop from a peak at 17 s (generated by the actinic light) compared to sample 1, while sample 1 reached a higher *F*_*s*_ and *F*_*m*_ *′* than the other samples. As a quantification of these differences, NPQ values were measured three times for each of these five samples (Table 1), demonstrating that NPQ varied significantly among samples from identical growth conditions. Similarly, Y(II) also differed significantly among the samples (Table 1). To observe NPQ as an indicator of RWC, we plotted the measured NPQ values against RWC for the *A. thaliana* samples (Figure 2A). The optimized regression of NPQ vs. RWC was linear and insignificant (*p* = 0.22) with an *R* ^2^ = 0.05.

**Figure 1:**
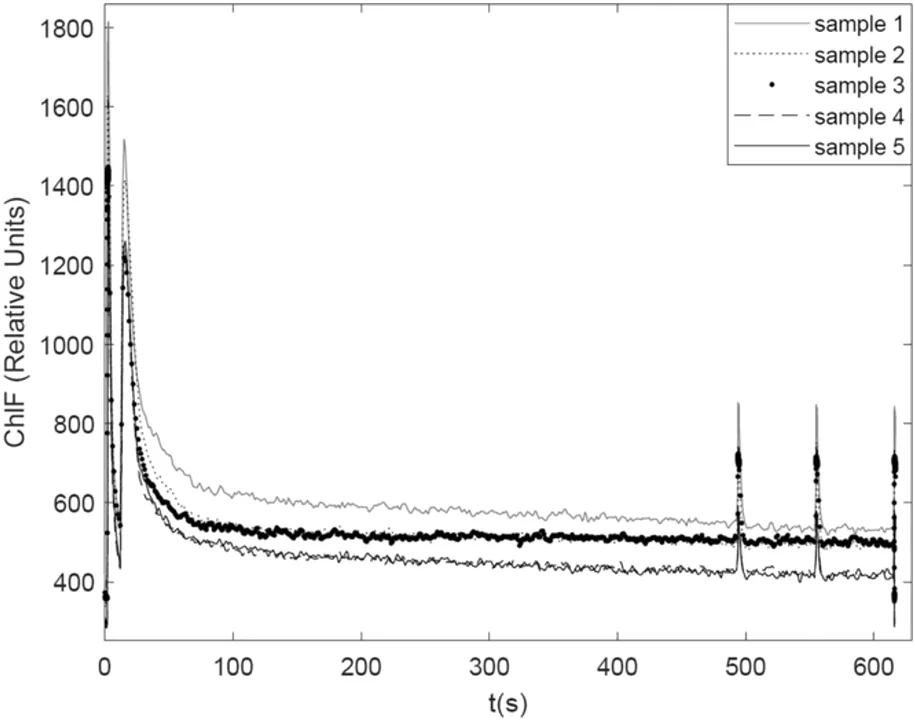
Chlorophyll fluorescence (ChlF) plots of detached, fresh, dark‐adapted *Arabidopsis thaliana* samples from identical growth conditions in response to a constant 1000‐μmol/m^2^/s actinic light (starting at 12 s) and saturating pulses (at 1.5, 493, 552, and 615 s). On the *x*‐axis, t signifies time (measured in seconds).

**Table 1:** Photosystem II (PSII) quantum efficiency in the light‐adapted state (Y(II)) and nonphotochemical quenching (NPQ) characteristics measured three times (*n* = 3) on each of five *Arabidopsis thaliana* plants grown in identical conditions shown in Figure 1.

	Sample plant no. (mean ± SD)				
PSII characteristic	1	2	3	4	5
Y(II)	0.30 ± 0.063^abc^	0.34 ± 0.018^a^	0.31 ± 0.002^b^	0.26 ± 0.021^c^	0.27 ± 0.011^c^
NPQ	1.23 ± 0.082^ab^	1.06 ± 0.092^a^	1.10 ± 0.075^a^	1.37 ± 0.085^bc^	1.40 ± 0.048^c^

*Note*: Different superscript letters indicate significant differences in a row at *p* < 0.05.

**Figure 2:**
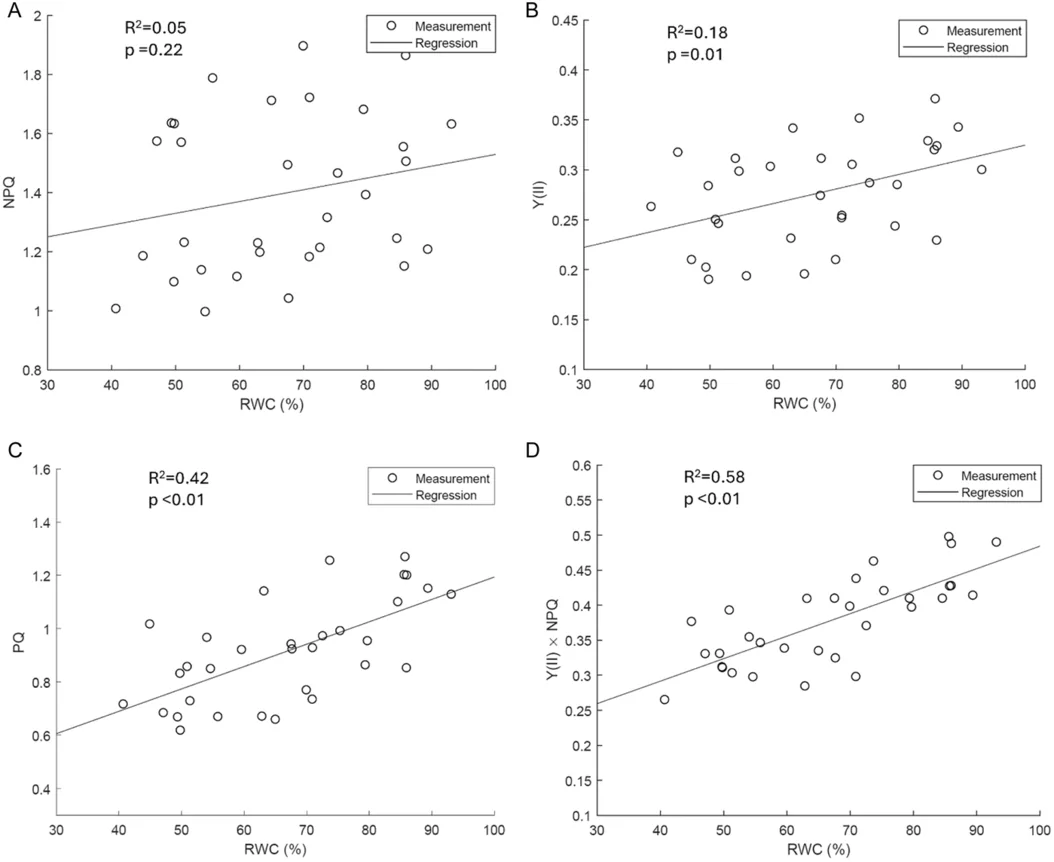
Regression analysis of (A) nonphotochemical quenching (NPQ), (B) photosystem II (PSII) quantum efficiency in the light‐adapted state (Y(II)), (C) photochemical quenching (PQ), and (D) NPQ‐scaled PSII quantum efficiency of photochemistry (Y(II)′ = Y(II) × NPQ) vs. relative water content (RWC) for *Arabidopsis thaliana* leaf samples (*n* = 5).

### Formulation of ChlF‐based indicators of RWC with reduced variability

Previous research has demonstrated that the photosynthetic quantum yield diminishes with decreasing RWC (Lu and Zhang, 1999). For the *A. thaliana* leaves, the PSII quantum efficiency under light, often symbolized as Y(II) or Φ_PSII_, declined significantly (*p* = 0.01) with increasing water loss (*R* ^2^ = 0.18, *p* = 0.01; Figure 2B), reflecting a discernible relationship between Y(II) and RWC. This shows that Y(II) is a viable candidate indicator of RWC and opportunities may exist to reduce its variability.

Because NPQ is a component of Y(II) and varies from sample to sample without a consistent relationship with RWC, as shown earlier, it adds variability to Y(II), making Y(II) a less‐effective indicator of RWC. This variability may therefore be reduced by eliminating or decreasing the influence of NPQ in a reformulated ChlF characteristic.

Y(II) is defined as a ratio, (*F*_*m*_ *′* − *F*_*s*_)/*F*_*m*_ *′*, from the measurable quantities *F*_*m*_ *′* and *F*_*s*_. As will be shown below (under “Analysis of other NPQ‐related ChlF characteristics”), there is a strong linear correlation between *F*_*s*_ and *F*_*m*_ *′*. We may thus replace *F*_*m*_ *′* in the denominator with *F*_*s*_ without fundamentally changing the correlation of the ratio with other variables. This alternative ratio, (*F*_*m*_ *′* − *F*_*s*_)/*F*_*s*_, can be simplified as follows:

(1) $$\frac{F_{m}\prime -F_{s}}{F_{s}}=\frac{\frac{k_{F}}{k_{F}+k_{N}+k_{D}\;}-\frac{k_{F}}{k_{F}+k_{N}+k_{P}+k_{D}}}{\frac{k_{F}}{k_{F}+k_{N}+k_{P}+k_{D}}}=\frac{\frac{k_{F}\left(k_{F}+k_{N}+k_{P}+k_{D}\right)-k_{F}\left(k_{F}+k_{N}+k_{D}\right)}{\left({k_{F}+k_{N}+k_{D})(k}_{F}+k_{N}+k_{P}+k_{D}\right)}}{\frac{k_{F}}{k_{F}+k_{N}+k_{P}+k_{D}}}=\frac{\frac{k_{F}k_{P}}{\left({k_{F}+k_{N}+k_{D})(k}_{F}+k_{N}+k_{P}+k_{D}\right)}}{\frac{k_{F}}{k_{F}+k_{N}+k_{P}+k_{D}}}=\frac{k_{P}}{k_{F}+k_{N}+k_{D}}=\frac{k_{P}}{\left(k_{F}+k_{D})(1+\text{NPQ}\right)}$$

where $F_{m}\prime =\frac{k_{F}}{k_{F}+{\;k}_{N}+{\;k}_{D}}$, $F_{s}=\frac{k_{F}}{k_{F}+k_{N}+k_{P}+k_{D}}$, and $\text{NPQ}=\frac{k_{N}}{k_{N}+k_{D}}$ have been applied; $k_{F}$, $k_{N}$, $k_{P}$, and $k_{D}$ represent the rate constants of fluorescence, nonphotochemical dissipation, photochemistry, and nonradiative dissipation, respectively.

Equation 1 shows that NPQ may be eliminated from the ratio by multiplying both sides by (1 + NPQ):

(2) $$\frac{F_{m}\prime -F_{s}}{F_{s}}\left(1+\text{NPQ}\right)=\frac{F_{m}\prime -F_{s}}{F_{s}}\left(1+\frac{F_{m}-F_{m}\prime }{F_{m}\prime }\right)=\frac{F_{m}}{F_{s}}-\frac{F_{m}}{F_{m}\prime }$$

Or alternatively,

(3) $$\frac{F_{m}\prime -F_{s}}{F_{s}}\left(1+\text{NPQ}\right)=\frac{k_{P}}{\left(k_{F}+k_{D}\right)\left(1+\text{NPQ}\right)}\left(1+\text{NPQ}\right)=\frac{k_{P}}{k_{F}+{\;k}_{D}}=\text{PQ}$$

Equations 2 and 3 show that eliminating NPQ from the Y(II)‐like ratio leads to $\frac{F_{m}}{F_{s}}-\frac{F_{m}}{F_{m}\prime }$, a measurable ChlF characteristic that is equal to $\frac{k_{P}}{k_{F}+k_{D}}$, the conventionally defined photochemical quenching (i.e., PQ) (Porcar‐Castell, 2011). Mechanistically, this is expected because water serves as the electron donor in PSII photochemistry. Water loss directly limits PSII photoelectron transport and photochemical activity, and thus this RWC‐dependent effect is reflected by PQ. Regression of PQ vs. RWC resulted in an *R* ^2^ value of 0.42 (*p* < 0.01) (Figure 2C). Without the influence of NPQ, PQ showed reduced scatter and thus increased *R* ^2^ value compared to Y(II) (Figure 2B).

Additionally, because there is a strong linear relationship between *F*_*s*_ and *F*_*m*_ *′* (shown below under “Analysis of other NPQ‐related ChlF characteristics”), Y(II) = (*F*_*m*_ *′* − *F*_*s*_)/*F*_*m*_ *′* should behave similarly to the alternative ratio (*F*_*m*_ *′* − *F*_*s*_)/*F*_*s*_ as discussed above. Because NPQ can be eliminated from the alternative ratio by multiplication with (1 + NPQ), the sample‐dependent NPQ effect in Y(II) should also be suppressed by multiplying with (1 + NPQ) or simply NPQ. As these differ by only a constant, we decided to test NPQ as a multiplier, which gave

(4) $$Y{\left(\text{II}\right)}^{\prime }=\text{NPQ}\times Y\left(\text{II}\right)=\frac{\left(F_{m}-F_{m}^{\;\prime }\right)\left(F_{m}^{\;\prime }-F_{s}\right)}{F_{m}^{\;\prime 2}}\;$$

Y(II)′ in Equation 4 may be referred to as an NPQ‐scaled PSII quantum efficiency of photochemistry. Experiments demonstrated that NPQ × Y(II) reduced scatter and increased *R* ^2^ value (*R* ^2^ = 0.58, *p* < 0.01; Figure 2D) compared with Y(II) alone. It should be noted that multiplication by NPQ does not make Y(II)′ a mechanistically accurate representation of PQ. Instead, Y(II)′ is a measure effectively scaled to reduce the influence of NPQ.

### Validation with different illumination intensities

Tests were performed to assess the dependence of Y(II), PQ, and Y(II)′ on RWC under different intensities of actinic light. In addition, qP = (*F*_*m*_ *′* − *F*)/(*F*_*m*_ *′* − *F*_0_′), which quantifies the fraction of open PSII reaction centers actively participating in photochemical reactions, was also measured. As illumination intensity increases, qP may diminish, indicating an elevated probability of reaction center closure.

Regressions of qP, Y(II), PQ, and Y(II)′ (= Y(II) × NPQ) vs. RWC for *A. thaliana* leaf samples under different actinic illumination intensities (750, 1250, and 1750 μmol/m^2^/s) are shown in Figure 3. First, qP did not vary with RWC for all three illumination intensities (*R* ^2^ < 0.1, *p* > 0.56), but the average qP value decreased from 0.66 to 0.48 to 0.41 as the illumination intensity increased from 750 to 1250 to 1750 μmol/m^2^/s, respectively. Second, qP values decreased with increasing illumination intensity (Figure 3A, E, I), aligning with findings from previous research (Porcar‐Castell, 2011), and Y(II) and Y(II)′ values were lower at higher illumination intensities (compare the vertical positions of these variables at different illumination intensities in Figure 3B, F, J and Figure 3D, H, L). These reductions point to a heightened probability of photoinhibition at higher illumination levels. PQ (Figure 3C, G, K) and Y(II)′ (= Y(II) × NPQ), nonetheless, resulted in greater regression *R* ^2^ values than Y(II) vs. RWC at all three illumination levels.

**Figure 3:**
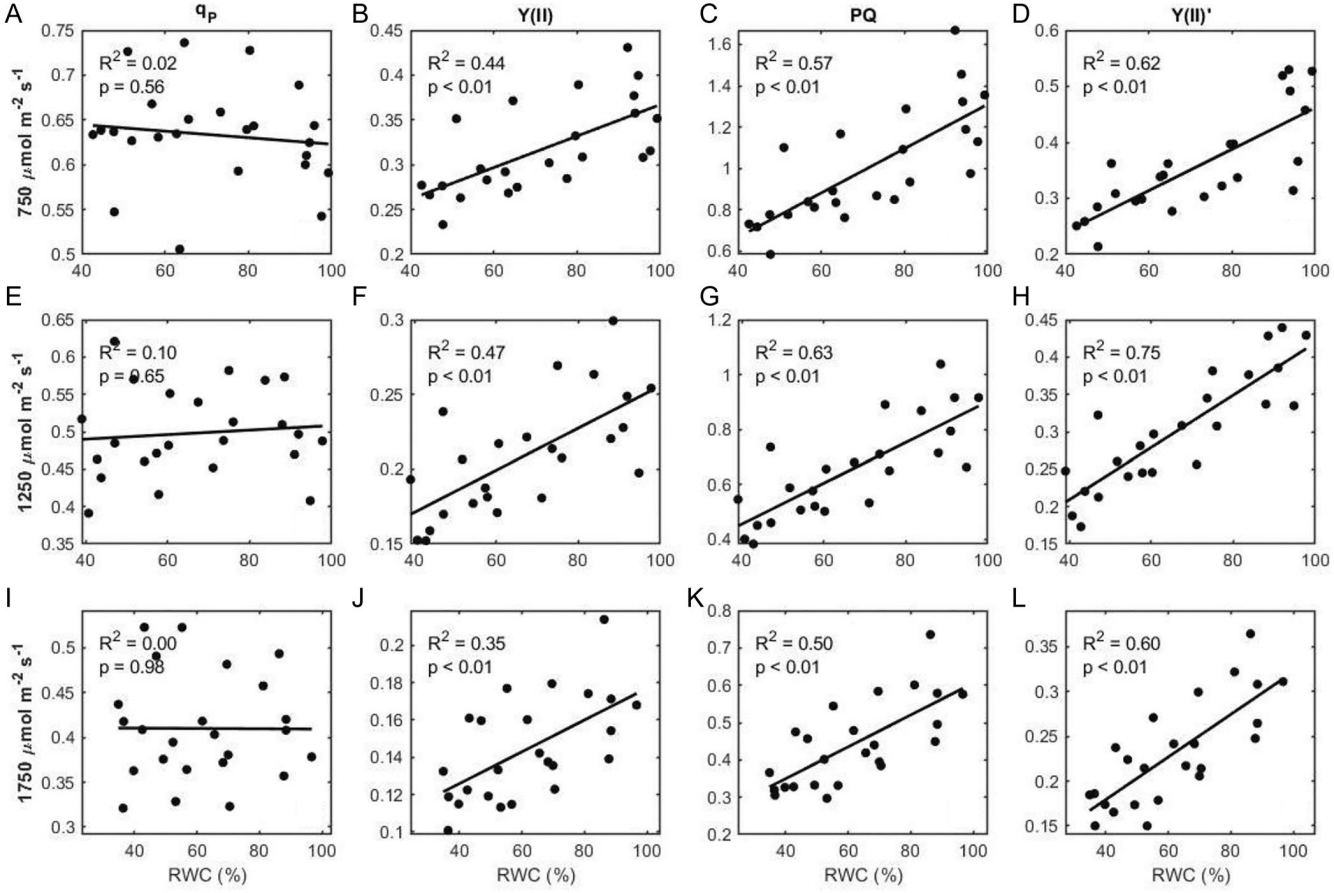
Regression analysis of photochemical capacity (qP), photosystem II (PSII) quantum efficiency in the light‐adapted state (Y(II)), photochemical quenching (PQ), and nonphotochemical quenching (NPQ)‐scaled PSII quantum efficiency of photochemistry (Y(II)′ = Y(II) × NPQ) vs. relative water content (RWC) for *Arabidopsis thaliana* leaf samples (*n* = 5) under actinic illumination intensities of (A–D) 750 μmol/m^2^/s, (E–H) 1250 μmol/m^2^/s, and (I–L) 1750 μmol/m^2^/s.

### Testing applicability of results obtained from *A. thaliana* in a different species

To test whether the concept demonstrated in *A. thaliana* also applies to other species, additional experiments were conducted with *S. oleracea*. Leaves from five plants were used to replicate experiments as described for *A. thaliana* and establish the relationships of NPQ, Y(II), PQ, and Y(II)′ to RWC in *S. oleracea*; the regression results of NPQ, Y(II), PQ, and Y(II)′ are presented in Figure 4. As seen previously, NPQ alone did not exhibit a significant correlation with RWC (*R* ^2^ = 0.047, *p* = 0.37; Figure 4A), whereas Y(II) showed a significant positive trend with RWC (*R* ^2^ = 0.56, *p* < 0.01; Figure 4B). Compared with Y(II), both PQ and Y(II)′ (= Y(II) × NPQ) led to stronger *R* ^2^ values (*R* ^2^ = 0.65, *p* < 0.01 [Figure 4C]; *R* ^2^ = 0.65, *p* < 0.01 [Figure 4D]; respectively). The repeatability of results from *A. thaliana* in *S. oleracea* suggests the potential for use of PQ or Y(II)′ to assess leaf RWC in different plant species.

**Figure 4:**
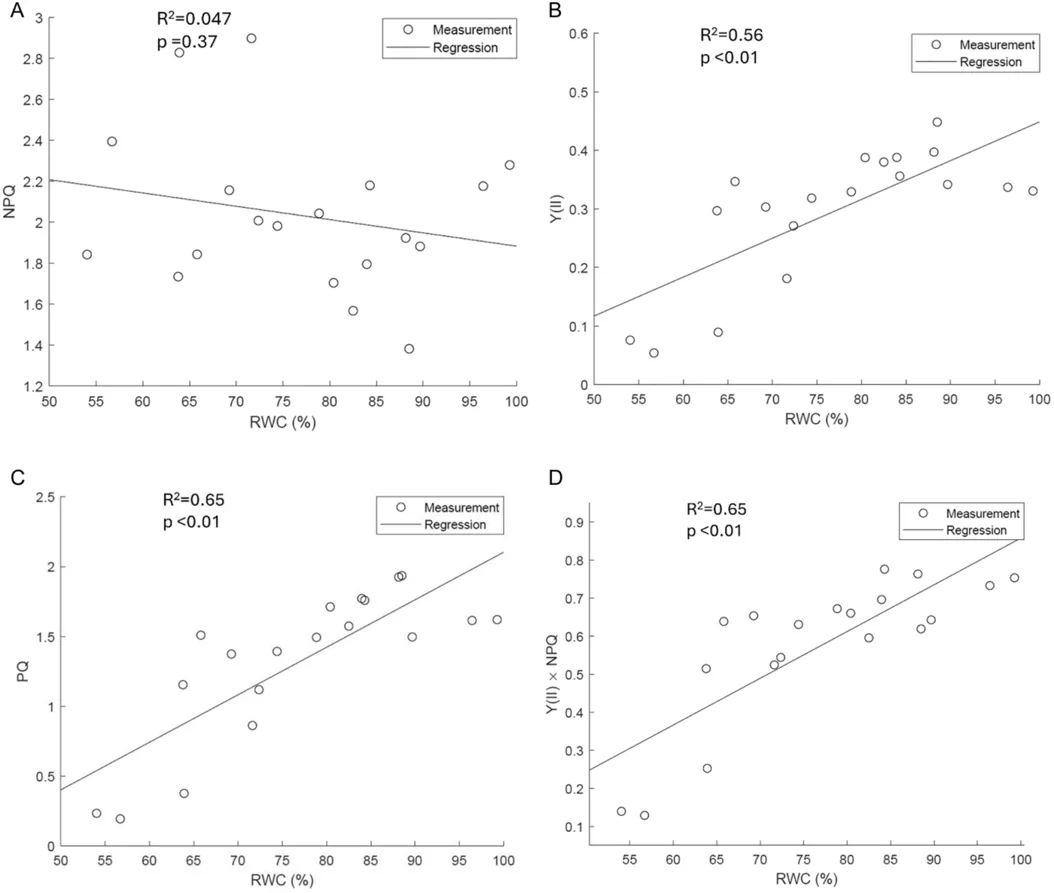
Regression analysis of (A) nonphotochemical quenching (NPQ), (B) photosystem II (PSII) quantum efficiency in the light‐adapted state (Y(II)), (C) photochemical quenching (PQ), and (D) NPQ‐scaled PSII quantum efficiency of photochemistry (Y(II)′ = Y(II) × NPQ) vs. relative water content (RWC) for *Spinacia oleracea* samples (*n* = 5).

### Analysis of other NPQ‐related ChlF characteristics

Because Y(II)′ depends on *F*_*m*_, *F*_*m*_ *′*, and *F*_*s*_ (Equation 4), which are all related to how ChlF descends from a peak after initiation of actinic light (at 17 s in Figure 1A) to a steady state, it is thus of interest to explore how these characteristic variables may correlate with one another. First, *F*_*m*_ *′* and *F*_*s*_ showed a linear positive correlation within each species during water loss, with a linear regression of *R* ^2^ ≥ 0.9 for both *A. thaliana* and *S. oleracea* (Figure 5). The slopes, nevertheless, were different (0.43 for *A. thaliana* and 0.81 for *S. oleracea*), indicating that the *F*_*s*_‐to‐*F*_*m*_ *′* relationship is robust within each species but the slope is species‐dependent. Remarkably, unlike NPQ, the correlation exhibited uniformity across replicated samples with strong *R* ^2^ (or *R*) values. The robust correlation between *F*_*m*_ *′* and *F*_*s*_ and the consistent RWC influence on both *F*_*m*_ *′* and *F*_*s*_ within a given species during light adaptation suggest a potential method for estimating NPQ when *F*_*m*_ *′* is unknown but *F*_*s*_ can be estimated.

**Figure 5:**
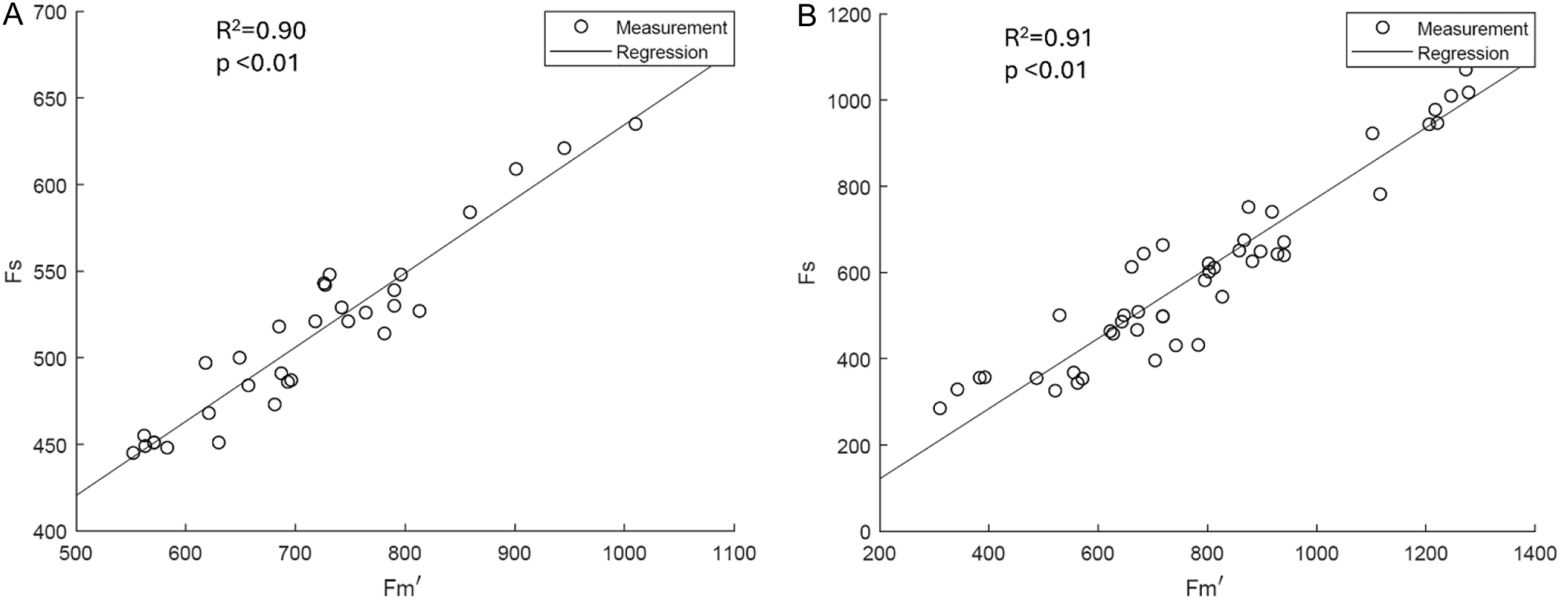
Regression of chlorophyll fluorescence (ChlF) in steady state (*F*_*s*_) vs. maximum ChlF in steady state (*F*_*m*_′) for (A) *Arabidopsis thaliana* (*n* = 5) and (B) *Spinacia oleracea* (*n* = 5).

Because *F*_*s*_ reflects the steady state of a photosynthetic system and the ChlF decline from a peak (Figure 1A) is largely affected by NPQ, the shape of the decline (often referred to as the PSMT phases) should be related to *F*_*s*_ and thus to *F*_*m*_ *′*, as a result of the correlation discussed above, and consequently related to NPQ. Although the ChlF decline during the PSMT phases is nonlinear and likely governed by multiple physiological processes, a first‐order approximation, as is often done in engineering analysis, is useful as a characterization of the dominant decay behavior. If the decline is approximated as a first‐order exponential curve, the dominant time constant (τ) can be used as a characteristic of the decline in ChlF. This time constant can be approximately measured from the ChlF curve, as shown in Figure 1, as the time required from the peak after actinic light starts to reach 63% of the total decay to *F*_*s*_, as described in the dynamic systems literature (e.g., Ogata, 2010). In this study, τ is not intended to represent a true single‐rate physiological process. Instead, it provides an effective time constant to represent how rapidly fluorescence decreases from the actinic‐light‐induced peak toward *F*_*s*_.

A modest but significant (*p* < 0.05) negative correlation between τ and NPQ is shown in Figure 6A, indicating an increase in τ as NPQ decreases. In other words, elevated NPQ is associated with an accelerated rate of decrease in ChlF and thus a smaller time constant in the PSMT phases (Figure 1). As a result, τ may be used to divide Y(II) to give an alternative form of Y(II)′ as:

(5) $${Y(\text{II})}_{\tau }^{\prime }=\frac{Y(\text{II})}{\tau }=\frac{F_{m}^{\;\prime }-F_{s}}{{\tau F}_{m}^{\;\prime }}\;$$

**Figure 6:**
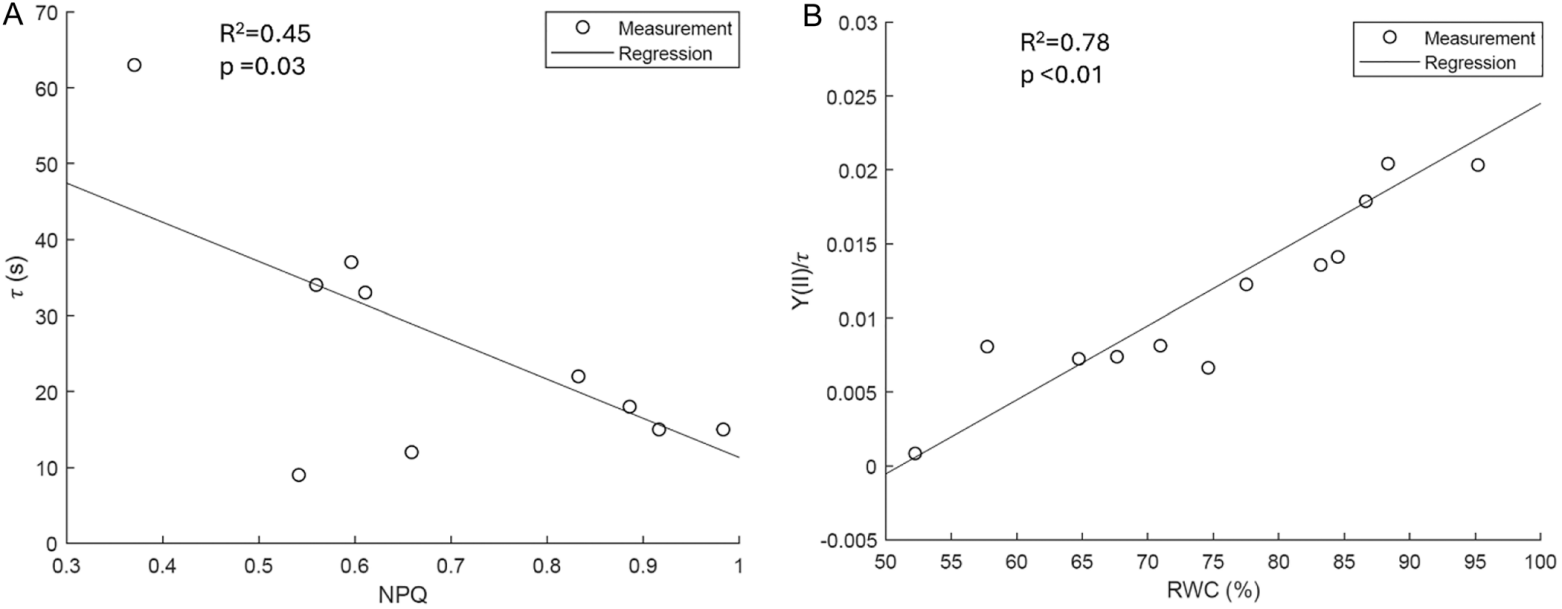
Regression analysis of (A) time constant (τ) vs. nonphotochemical quenching (NPQ), and (B) PSII quantum efficiency in the light‐adapted state divided by the dominant time constant (Y(II)/τ) vs. relative water content (RWC) for *Spinacia oleracea*.

The alternative form, ${Y(\text{II})}_{\tau }^{\prime }$, employing τ as a modifier of the PSII quantum efficiency, also resulted in a reduction in scatter and notable improvement in *R* ^2^ for regression against RWC (from 0.45 to 0.78) (Figure 6B), although τ is only an approximation with modest correction with NPQ. This improvement further substantiates our theory that reducing the effect of NPQ, even partially or approximately, improves the assessment of leaf RWC based on ChlF measurements.

## DISCUSSION

These results demonstrate that there can be considerable variations in NPQ measured from plants grown at the same time and under the same conditions, reducing the usefulness of ChlF characteristics as indicators of leaf RWC. As seen above, leaf RWC influenced NPQ, but the reductions in NPQ in some samples began at around 90% RWC, whereas others showed an initial decline at a RWC as low as 60%. This observation aligns with field measurements showing that NPQ decreases coincided with five days of drought and RWC levels were approximately 60% to 80% (Jia et al., 2020). Jia et al. (2020) found that while both *F*_*m*_ and *F*_*m*_ *′* decreased during leaf moisture loss, the maximum potential quantum efficiency remained relatively consistent (i.e., negligible *F*_*m*_ reduction) during short‐term drought but subsequently declined with prolonged drought. Additionally, Y(II) and NPQ initially exhibited opposite trends followed by a concurrent reduction over time (Singh et al., 2014). There was not a clear inflection point in variations in NPQ vs. time of drought. Furthermore, the correlation between NPQ and RWC was not robust because of inconsistencies (Jia et al., 2020), implying that the correlation between NPQ and RWC is contingent upon individual leaf characteristics. These inconsistencies in the relationship between NPQ and RWC indicate that NPQ by itself is not a good indicator of RWC of plants.

Other research corroborates these observed inconsistencies; for example, major variabilities in ChlF behavior have been found to be associated with nitrogen or pigment content (Hallik et al., 2012; Lacour et al., 2020), leaf anatomical characteristics (Johnson et al., 2005), and optical properties (Vogelmann and Evans, 2002). Variations in palisade mesophyll thickness, chloroplast packing density, chloroplast distribution, and optical path length through the palisade layer can alter the effective absorption cross sections of PSII and modify the heat‐dissipation rate constant *k*_*N*_. As a result, both the magnitude and kinetics of NPQ can vary substantially across samples because of these anatomical and other differences, causing raw NPQ to fluctuate unpredictably among replicates.

The leaf developmental stage is a known determinant of palisade mesophyll thickness, chloroplast density, pigment concentration, and xanthophyll cycle capacity (e.g., Hoshino et al., 2019). A lack of uniformity in sample developmental stage thus influences *k*_*N*_ and NPQ. During field measurement, differences among replicates are usually minimized by using leaf samples of similar size, color, and position, as was done in this work; however, there is no assurance of leaf consistency in terms of developmental measures such as the plastochron index and leaf plastochron index. This is another potential source of the sample‐to‐sample NPQ variability observed. While such a source of variation may be suppressed by more methodical leaf selection, a useful and convenient measurement method should be robust without relying on stringent measurement protocols.

To address these challenges, it is necessary to explore methods capable of mitigating the impact of NPQ variations among replicate samples. Using NPQ, either conventionally measured or indicated by another related variable (such as a major time constant), to formulate NPQ‐scaled/adjusted PSII quantum efficiency measures, such as PQ, Y(II)′, and ${Y(\text{II})}_{\tau }^{\prime }$, resulted in reduced variability under light and improved correlation with RWC.

This work revealed that qP exhibited minimal correlation with RWC, although it decreased with increasing radiation levels (Ruban and Murchie, 2012), suggesting that the extent of photoinhibition was largely unaffected by RWC. This underscores the viability of utilizing Y(II)′ or ${Y(\text{II})}_{\tau }^{\prime }$ as an indicator of RWC under varied illumination intensities. In addition, the derived NPQ‐independent measure corresponds with the definition of PQ. Given that the rate of photochemistry is inherently influenced by water content, a positive correlation between PQ and RWC is expected.

There was a strong linear correlation between *F*_*s*_ and *F*_*m*_ *′*, but the slope of the linear relationship differed between the two species studied here. This slope difference may be associated with anatomical traits specific to each species, such as palisade mesophyll thickness, chloroplast density, or pigment distribution. A thicker palisade layer or higher chloroplast density may increase internal light scattering and alter PSII excitation, thereby affecting the relative changes in *F*_*s*_ and *F*_*m*_ *′* (Vogelmann and Han, 2000; Borsuk and Brodersen, 2019). These anatomical and optical differences may explain the steeper slope observed for *S. oleracea* than for *A. thaliana*.

It should be noted that the formulation in Equation 5 may not be optimal, as the time constant used in the equation is only a rudimentary characterization of NPQ‐induced kinetic variations in ChlF. Interestingly, while the formulation of Y(II)′ in Equation 4 represents an approximate cancellation of NPQ effects, the *R* ^2^ values for Y(II)′ were no worse (and were actually higher in most cases) than those for PQ as an indicator of RWC. The exact reason for the apparent advantage of Y(II)′ is not clear and thus such an advantage is not assured. An understanding of the causes for NPQ variations and how they affect PSII quantum efficiency is necessary for further refinement and understanding of the formulations.

The results from this work support and are also corroborated by the findings from our earlier work based on dynamic analysis in the frequency domain of ChlF (Chen et al., 2024). In the earlier work, we reported that the low‐frequency gain, which is a characterization of the slow ChlF decline under a constant actinic light, has a strong negative correlation with NPQ, whereas the high‐frequency gain has significant correlations with RWC. The high‐frequency gain is the ratio of the amplitude of ChlF response (similar to *F*_*m*_ *′* − *F*_*s*_) over that of a fast‐varying excitation such as a pulse and is thus an indicator of photochemical efficiency, similar to Y(II). Subtracting the low‐frequency gain from the high‐frequency gain to remove or reduce the effect of NPQ variations improved the *R* ^2^ values for regression vs. RWC for five different plants, including *S. oleracea*, *A. thaliana*, and woody plants. Because gain values calculated in the frequency domain are expressed in decibels, which involves a logarithmic operation, subtracting the low‐frequency gain is equivalent to division by the gain without the logarithmic unit conversion or to multiplication (because of the negative correlation) by NPQ, as done in this work.

The present study tested the proposed NPQ‐adjusted ChlF measures by using two C_3_ eudicot species, *A. thaliana* and *S. oleracea*. The rate‐constant‐based analysis to arrive at PQ and Y(II)′ is generally applicable because a water deficit primarily influences PSII photochemistry in C_3_ plants. Responses to water deficit by some C_4_ grasses, however, may be dominated by stomatal limitation and CO_2_ availability rather than direct impairment of PSII photochemistry (Da Silva and Arrabaca, 2004). In addition, in C_4_ species, Kranz anatomy, spatial separation of light harvesting and carbon fixation, differences in chloroplast distribution, and altered xanthophyll‐cycle activity can change the relative contributions between photochemical and nonphotochemical energy dissipation (Liu et al., 2022). Therefore, further validations are needed to extend the applicability of these proposed indicators to C_4_ plants.

It should also be noted that the dehydration protocol used in this study differs from soil‐mediated drought in intact plants. In this work, excised leaves were allowed to lose water under controlled room conditions to generate a broad range of RWC values for evaluating the relationship between ChlF characteristics and leaf water status. This approach reduces environmental variability and enables direct comparison among samples, but it does not fully reproduce the physiological complexity of drought development in intact plants. Under soil‐mediated drought, water loss is typically more gradual and is accompanied by root‐to‐shoot signaling, stomatal regulation, and changes in gas exchange. These processes can alter NPQ induction, PSII photochemical activity, and the kinetics of fluorescence changes. Therefore, the present results should be interpreted as a demonstration of the effectiveness of NPQ‐adjusted ChlF measures under controlled leaf dehydration conditions. Further testing under intact‐plant and field drought conditions is needed to determine how broadly these metrics can be applied for drought assessment.

### Concluding remarks

In summary, this research shows that NPQ changes ChlF measurements in the light‐adapted state and that scaling ChlF characteristics by NPQ enhances their usefulness as indicators of plant leaf relative water content. The results provide a potential method for developing useful and convenient ChlF‐based indicators of drought. Further field testing of these characteristics as drought indicators is warranted.

## AUTHOR CONTRIBUTIONS

J.C. did experiments, data analysis, programming, and writing of the original draft; L.F. participated in data analysis and validation; Y.G., F.B.F., and J.T. conceived the project, acquired funding, supervised the work, and edited the manuscript. All authors reviewed and approved the final manuscript.

## Data Availability

The datasets generated during and/or analyzed during the current study are available at https://github.com/Sophiecjq/ChlF-characteristics-matlab-with-data.
